# Supplementary material for: Inhibition of hypoxia‐inducible factors suppresses subretinal fibrosis
Source: FASEB J. 2024 Jul 2;38(13):e23792. doi: 10.1096/fj.202400540RRR (PMC13688073; doi:10.1096/fj.202400540RRR)
Supplement: Supplementary file 1 [file FSB2-38-e23792-s001.docx]

| **Table S2.** Immunohistochemical analyses in AMD | | | |  |
| --- | --- | --- | --- | --- |
| **Diagnosis** | **Age (yr) / Gender / Eye** | **Preoperative visual acuity (LogMAR)** | **Expression (strong +/ weak ± / none -)** | |
| PCV | 72 / Male / Right | 1.0 | HIF-1α (+), HIF-2α (±), ZEB2 (+), Collagen I α1 (+) | |
| PCV | 74 / Male / Left | 0.4 | HIF-1α (+), HIF-2α (+), ZEB2 (+), Collagen I α1 (+) | |
| PCV | 76 / Male / Left | 0.2 | HIF-1α (+), HIF-2α (+), ZEB2 (+), Collagen I α1 (+) | |
| Type 2 MNV | 58 / Male / Left | 0.5 | HIF-1α (+), HIF-2α (±), ZEB2 (+), Collagen I α1 (+) | |
| PCV | 75 / Male / Right | 0.6 | HIF-1α (+), HIF-2α (-), ZEB2 (+), Collagen I α1 (+) | |
