# Supplementary material for: Inhibition of hypoxia‐inducible factors suppresses subretinal fibrosis
Source: FASEB J. 2024 Jul 2;38(13):e23792. doi: 10.1096/fj.202400540RRR (PMC13688073; doi:10.1096/fj.202400540RRR)
Supplement: Supplementary file 2 [file FSB2-38-e23792-s002.docx]

| **Table S3.** Compositions of histidine and taurine in our marine products | | |
| --- | --- | --- |
| **Fish** | **Histidine (mg/100 g FW)** | **Taurine (mg/100 g FW)** |
| *D.tabl* | 118.0 | 473.6 |
| *D.muroadsi* | 95.2 | 432.1 |
| FW, fresh weight. | | |
