# Supplementary material for: Inhibition of hypoxia‐inducible factors suppresses subretinal fibrosis
Source: FASEB J. 2024 Jul 2;38(13):e23792. doi: 10.1096/fj.202400540RRR (PMC13688073; doi:10.1096/fj.202400540RRR)
Supplement: Supplementary file 3 [file FSB2-38-e23792-s003.docx]

**Figure S3.** Suppression of retinal neovascularization in a murine model of oxygen-induced retinopathy (OIR) by treatment of histidine or taurine. **(A and B)** Images and quantitative data (n = 6 per group) showed that neovascular tufts (red) were reduced by treatment with histidine (His) or taurine (Tau), while vaso-obliteration (yellow) was not changed. *P <0.05. Two-tailed Student’s *t*-test. Boxplots were presented as median with interquartile range, the 25th and 75th percentile.
