# Supplementary material for: Inhibition of hypoxia‐inducible factors suppresses subretinal fibrosis
Source: FASEB J. 2024 Jul 2;38(13):e23792. doi: 10.1096/fj.202400540RRR (PMC13688073; doi:10.1096/fj.202400540RRR)
Supplement: Supplementary file 4 [file FSB2-38-e23792-s004.docx]

**Figure S1.** PCR results of conditional knockout (cKO) of genes used in the current study. Best1-Vhl, Best1-Hif1 and Hif2, Best1-Hif1, and Best1-Hif2 PCR images are presented. Primer sequences used for this work are as follows: Best1-Cre forward (F): ATG CCC AAG AAG AAG AGG AAG GTG TCC, Best1-Cre Reverse (R): TGG CCC AAA TGT TGC TGG ATA GTT TTT A, Hif1 F: AAA ATG TAG TCC TGT TGG TTG TGC, Hif1 R: GGC AGT ACT GGA AAG ATG GCT AAG, Hif2 F: TCC TGG AAG CCC ATC CAG TAC CTC, Hif2 R: CAG GCA GTA TGC CTG GCT AAT TCC AGT T, Vhl F: CTA GGC ACC GAG CTT AGA GGT TTG CG, Vhl R: CTG ACT TCC ACT GAT GCT TGT CAC AG. Sequencing information was referenced as follows (https://www.jax.org/): Best1-Cre (RRID:IMSR_JAX:017557), Hif1 (RRID:IMSR_JAX:007561) , Hif2 (RRID:IMSR_JAX:008407), Vhl: (RRID:IMSR_JAX:012933).
