# Supplementary material for: Inhibition of hypoxia‐inducible factors suppresses subretinal fibrosis
Source: FASEB J. 2024 Jul 2;38(13):e23792. doi: 10.1096/fj.202400540RRR (PMC13688073; doi:10.1096/fj.202400540RRR)
Supplement: Supplementary file 5 [file FSB2-38-e23792-s005.docx]

| **Table S1.** Primer list | | |  |
| --- | --- | --- | --- |
| **Name** | **Direction** | **Sequence (5' → 3')** | |
| *ZEB1* | Forward | TTACACCTTTGCATACAGAACCC | |
|  | Reverse | TTTACGATTACACCCAGACTGC | |
| *ZEB2* | Forward | GCGATGGTCATGCAGTCAG | |
|  | Reverse | CAGGTGGCAGGTCATTTTCTT | |
| *COL1A1* | Forward | GAGGGCCAAGACGAAGACATC | |
|  | Reverse | CAGATCACGTCATCGCACAAC | |
| *GAPDH* | Forward | TCCCTGAGCTGAACGGGAAG | |
|  | Reverse | GGAGGAGTGGGTGTCGCTGT | |
| *Zeb1* | Forward | GCTGGCAAGACAACGTGAAAG | |
|  | Reverse | GCCTCAGGATAAATGACGGC | |
| *Zeb2* | Forward | CCACGCAGTGAGCATCGAA | |
|  | Reverse | CAGGTGGCAGGTCATTTTCTT | |
| *Gapdh* | Forward | AGGAGCGAGACCCCACTAAC | |
|  | Reverse | GATGACCCTTTTGGCTCCAC | |
