# Supplementary material for: Inhibition of hypoxia‐inducible factors suppresses subretinal fibrosis
Source: FASEB J. 2024 Jul 2;38(13):e23792. doi: 10.1096/fj.202400540RRR (PMC13688073; doi:10.1096/fj.202400540RRR)
Supplement: Supplementary file 6 [file FSB2-38-e23792-s006.docx]

**Figure S2.** 2D images of choroidal neovascularization and subretinal fibrosis for the current study’s reference. 2D representative images are additionally presented for each experiment, while the 3D images are shown in the main article.
